# Differential Effects of CORM-2 and CORM-401 in Murine Intestinal Epithelial MODE-K Cells under Oxidative Stress

**DOI:** 10.3389/fphar.2017.00031

**Published:** 2017-02-08

**Authors:** Dinesh Babu, Georges Leclercq, Roberto Motterlini, Romain A. Lefebvre

**Affiliations:** ^1^Heymans Institute of Pharmacology, Faculty of Medicine and Health Sciences, Ghent UniversityGhent, Belgium; ^2^Department of Clinical Chemistry, Microbiology and Immunology, Faculty of Medicine and Health Sciences, Ghent UniversityGhent, Belgium; ^3^INSERM U955, Faculty of Medicine, Equipe 12 and University Paris EstCréteil, France

**Keywords:** carbon monoxide-releasing molecules, hydrogen peroxide, intestinal epithelial cells, mitochondria, oxidative stress, reactive oxygen species, solubility, TNF-α/CHX

## Abstract

Carbon monoxide (CO)-releasing molecules (CO-RMs) are intensively studied to provide cytoprotective and anti-inflammatory effects of CO in inflammatory conditions including intestinal inflammation. The water-soluble CORM-A1 reduced apoptosis and NADPH oxidase (NOX)-derived reactive oxygen species (ROS) induced by tumor necrosis factor (TNF)-α/cycloheximide (CHX) in mouse MODE-K intestinal epithelial cells (IECs), without influencing TNF-α/CHX-induced mitochondrial superoxide anion ($O_{2}^{•–}$). The aim of the present study in the same model was to comparatively investigate the influence of lipid-soluble CORM-2 and water-soluble CORM-401, shown *in vitro* to release more CO under oxidative conditions. CORM-2 abolished TNF-α/CHX-induced total cellular ROS whereas CORM-401 partially reduced it, both partially reducing TNF-α/CHX-induced cell death. Only CORM-2 increased mitochondrial $O_{2}^{•–}$ production after 2 h of incubation. CORM-2 reduced TNF-α/CHX-, rotenone- and antimycin-A-induced mitochondrial $O_{2}^{•–}$ production; CORM-401 only reduced the effect of antimycin-A. Co-treatment with CORM-401 during 1 h exposure to H_2_O_2_ reduced H_2_O_2_ (7.5 mM)-induced ROS production and cell death, whereas CORM-2 did not. The study illustrates the importance of the chemical characteristics of different CO-RMs. The lipid solubility of CORM-2 might contribute to its interference with TNF-α/CHX-induced mitochondrial ROS signaling, at least in mouse IECs. CORM-401 is more effective than other CO-RMs under H_2_O_2_-induced oxidative stress conditions.

## Introduction

Acute and chronic gastrointestinal (GI) inflammatory disease conditions are associated with persistent oxidative stress originating from increased reactive oxygen species (ROS) that are known to initiate and perpetuate inflammation (Bhattacharyya et al., 2014; Mittal et al., 2014). Oxidative stress-induced epithelial cell damage and increased intestinal permeability toward luminal commensal bacterial material, which activates mucosal immune cells and triggers muscular inflammation, can contribute to the severity of acute GI disorders as observed in animal models of septic ileus, necrotizing enterocolitis and ischemia/reperfusion injury (Anup et al., 1999; de Winter et al., 2005; Baregamian et al., 2009; De Backer et al., 2009; Guan et al., 2009). Excessive production of ROS and mucosal injury has also been reported in animal models of inflammatory bowel disease (IBD) (Ahn et al., 2001; Reifen et al., 2004; Cetinkaya et al., 2005) and in colonic tissue of patients with ulcerative colitis (Oshitani et al., 1993; Nishikawa et al., 2005). Tumor necrosis factor (TNF)-α is one of the early inflammatory mediators thought to play an important role in epithelial barrier dysfunction by inducing intestinal epithelial cell (IEC) apoptosis. ROS play an important role in TNF-α-induced apoptotic cell death of IECs (Jin et al., 2008; Baregamian et al., 2009; Babu et al., 2012). The nicotinamide adenine dinucleotide phosphate (NADPH) oxidase (NOX) family and the mitochondrial electron transport chain (ETC) are the two major ROS-producing sources involved in TNF-α/cycloheximide (CHX)-induced cell death in rat IEC-6 cells (Jin et al., 2008) and in mouse MODE-K cells (Babu et al., 2015b). In MODE-K cells in particular, complexes I and II of the mitochondrial ETC were found to be the main sites of superoxide anion ($O_{2}^{•–}$) production in addition to NOX (Babu et al., 2015b). As the endogenous antioxidant defense system does not seem sufficient to counteract TNF-α-induced ROS production, neutralizing excessive ROS production might be an effective therapeutic strategy to reduce intestinal barrier dysfunction during GI inflammation.

The stress-responsive protein heme oxygenase (HO)-1 is up-regulated by oxidative stress and inflammatory signals, and it generates biliverdin, a powerful antioxidant, and carbon monoxide (CO), which exerts antioxidant, anti-inflammatory and cytoprotective effects (Ryter et al., 2006; Motterlini and Otterbein, 2010). Inhalation of CO gas has been successfully applied in animal models of inflammation and oxidative stress but translation to humans might be difficult (Ji et al., 2016). From pharmacological and therapeutic perspectives, small molecules capable of delivering controlled amounts of CO to biological systems have therefore been developed to mimic the intrinsic beneficial effects of CO (Motterlini et al., 2002; Sawle et al., 2005; Motterlini and Otterbein, 2010). These compounds, known as CO-releasing molecules (CO-RMs), have been extensively studied and belong to two major classes: (1) metal carbonyl complexes containing ruthenium, manganese, or molybdenum, which carry CO bound to the transition metal and (2) boranocarbonates, which do not contain transition metals but the metalloid boron and release CO spontaneously in physiological conditions, the rate of release being affected by changes in pH. While the original lipophilic CO-RMs such as CORM-1 ([Mn_2_(CO)_10_]) and CORM-2 ([Ru(CO)_3_Cl_2_]_2_) have to be dissolved in organic solvents such as dimethyl sulfoxide (DMSO) (Motterlini et al., 2002), water-soluble CO-RMs such as CORM-3 ([Ru(CO)_3_Cl(glycinate)]) and CORM-A1 (Na_2_[H_3_BCO_2_]) were subsequently developed (Clark et al., 2003; Motterlini et al., 2005b). These compounds have been shown to be pharmacologically active in limiting cellular and tissue dysfunctions in a number of pathological disorders associated with inflammation and tissue injury (Motterlini and Foresti, 2014). The inhibitory effect of CO and CO-RMs on cytokine-induced changes in the intestinal epithelium might also contribute to their beneficial effect in acute GI inflammation such as postoperative ileus (Babu et al., 2015c) and in chronic GI inflammation such as IBD (Takagi et al., 2015). Although the exact mechanism(s) for the antioxidant and cytoprotective effect of CO is (are) still under investigation, emerging evidence indicates that the beneficial properties of CO may be linked to its ability to bind to hemoproteins, such as NOX and mitochondrial complexes in different tissues (Taille et al., 2005; Bilban et al., 2008). At the mitochondrial level, CO was shown to induce a transient burst of mitochondrial ROS ($O_{2}^{•–}$) that is thought to promote a preconditioning state, enabling it to counteract subsequent oxidative stress (Taille et al., 2005; Chin et al., 2007; Vieira et al., 2008). We previously showed that the water-soluble CORM-A1 reduced both TNF-α/CHX-induced ROS production and apoptosis in MODE-K IECs (Babu et al., 2012, 2015a). At cytoprotective concentrations, CORM-A1 *per se* did not induce mitochondrial $O_{2}^{•–}$; however, CORM-A1 inhibited NOX-derived ROS production, but not mitochondrial $O_{2}^{•–}$ production, after challenging MODE-K IECs with TNF-α (Babu et al., 2015a). This absence of an effect at the mitochondrial level might be related to the water-soluble properties of this CO-RM, which prevent its penetration to sites of ROS production in mitochondria. By contrast, the lipophilic CORM-2 was shown to induce ROS production from mitochondria in human bronchial smooth muscle cells (Taille et al., 2005). The cytoprotective properties of CORM-2 in IECs and its effect on cellular targets mediating ROS production have yet to be investigated. CORM-401 [Mn(CO)_4_{S_2_CNMe(CH_2_CO_2_H)}] is a recently developed water-soluble CO-RM that releases up to three equivalents of CO per mole of compound, in contrast to CORM-A1 which releases one equivalent of CO (Motterlini et al., 2005a; Crook et al., 2011; Fayad-Kobeissi et al., 2016). Moreover, the rate of CO release from CORM-401 in cell-free *in vitro* systems is accelerated in the presence of biologically relevant oxidants, such as hydrogen peroxide (H_2_O_2_) (Fayad-Kobeissi et al., 2016).

In view of the above considerations, in the present study, we compared the cytoprotective effects of CORM-2 and CORM-401 in MODE-K IECs under oxidative stress conditions, evaluating their effects on oxidant-generating system(s). For the protocol with a high concentration of H_2_O_2_, also CORM-A1 was compared as this was not investigated in our previous study with the compound (Babu et al., 2015a). The major characteristics of the three CO-RMs are summarized in **Table 1**.

**Table 1:** Structure and characteristics of CO-RMs studied.

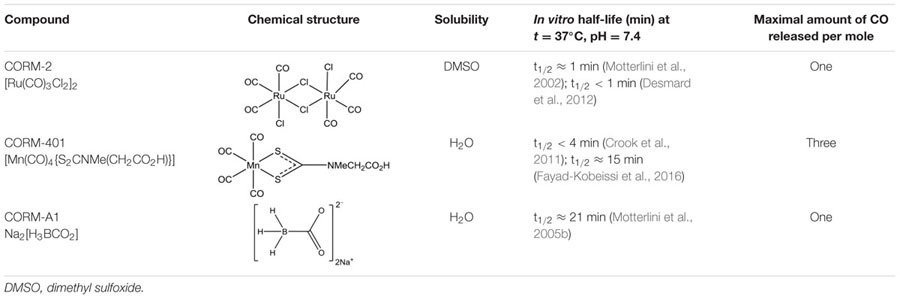

## Materials and Methods

### Chemicals and Reagents

Reagents for cell culture, including Dulbecco’s modified Eagle’s medium (DMEM), fetal bovine serum, penicillin/streptomycin and GlutaMAX were obtained from Gibco BRL (Grand Island, NY, USA). Carboxylated analog of 2′7′-dichlorodihydrofluorescein diacetate acetyl ester (carboxy-H_2_DCFDA), MitoTracker Deep Red FM, MitoTracker Green FM, MitoSOX Red, Sytox Green, Sytox Red and tetramethylrhodamine methyl ester (TMRM) were purchased from Molecular Probes – Invitrogen (Carlsbad, CA, USA). Recombinant murine TNF-α was purchased from R&D systems (Minneapolis, MN, USA). Antimycin-A, CHX, CORM-2, DMSO, H_2_O_2_ and rotenone were purchased from Sigma (St. Louis, MO, USA). CORM-A1 and CORM-401 were synthesized as previously described (Motterlini et al., 2005b; Crook et al., 2011). Stock solutions of CORM-2 were prepared in DMSO, whereas CORM-401 was dissolved in phosphate buffered saline (PBS); the solutions were both protected from light in all experiments. Inactive CORM-2 (iCORM-2) was prepared by keeping the stock solution in DMSO for 18 h at 37°C in a 5% CO_2_ humidified atmosphere to liberate CO (Sun et al., 2008). As CORM-401 solutions as such are stable, no iCORM-401 comparable to iCORM-2 can be prepared. Fayad-Kobeissi et al. (2016) used a mixture of equimolar amounts of Na_2_CNMeCO_2_Na.nH_2_O and MnSO_4_ as a surrogate iCORM-401 as these compounds compose the skeletal basis of the CORM-401 molecule without CO; this mixture did not reproduce the effects of CORM-401 in the *in vitro* models of vascular relaxation and angiogenesis studied. H_2_O_2_, obtained in a liquid formulation, was dissolved in culture medium. All other chemicals were dissolved in DMSO, except TNF-α, which was dissolved in PBS.

### Cell Culture

The mouse small IEC line, MODE-K (a generous gift from Dr. Ingo B. Autenrieth, University of *Tübingen*, Germany) was used in our study. This cell line was derived from the duodenum-jejunum from normal young C3H/HeJ mouse immortalized by simian virus (SV)-40 large T gene transfer. The cells are undifferentiated but still exhibit morphological and phenotypic characteristics of normal enterocytes (Vidal et al., 1993). MODE-K cells (passage 10–35) were cultured in DMEM medium supplemented with 10% fetal bovine serum, 2 mM L-glutamine, and 5% penicillin (10,000 units/ml) with streptomycin (10 mg/ml). Cultures were maintained in a humidified 5% CO_2_ atmosphere at 37°C, and experiments were conducted on cells at approximately 80–90% confluence. MODE-K cells were seeded in culture medium containing 10% serum, grown for 36 h, and then serum-starved overnight. Cells were seeded at either 1 × 10^4^ cells per 200 μL of culture medium per well in a 96-well microtiter plate (for cell viability assay) or 2.5 × 10^5^ cells per 2 mL of culture medium per well in a six-well plate (for all other assays).

### Treatment Protocols

At 48 h after seeding, MODE-K cells were exposed to oxidative stress by treatment with 1 ng/ml TNF-α plus 10 μg/ml CHX for 6 h, 1 mM H_2_O_2_ for 40 min, 7.5 mM H_2_O_2_ for 1 h, 7.5 μM rotenone for 6 h, or 10 μM antimycin-A for 6 h. To study the influence of CORM-2 and CORM-401 vs. the oxidant stimuli, CO-RMs were pre-incubated from 1 h before exposure to the oxidative stress stimulus, followed by co-incubation of CO-RMs with the stimulus. However, for the experiments involving 7.5 mM H_2_O_2_ for 1 h, CORM-2 and CORM-401 were also just pre-incubated for 1 h or only co-incubated for 1 h; CORM-A1 was also studied vs. 7.5 mM H_2_O_2_, as this was not performed in the previous study on CORM-A1 (Babu et al., 2015a).

CORM-2 and CORM-401 were used at 40 and 50 μM, respectively, as these were the highest concentrations without an effect *per se* on cell viability of MODE-K cells when incubated for 12 h (*n* = 3 for each compound, data not shown). CORM-A1 was studied at 100 μM, as determined earlier with the same assay (Babu et al., 2012, 2015a).

### Determination of Cell Viability

Cell viability was assessed by luminescent cell viability assay with CellTiter-Glo (Promega, Madison, WI, USA) according to the manufacturer’s protocol. This assay determines the number of viable cells in culture based on quantitation of ATP, an indicator of metabolically active cells. Briefly, MODE-K cells were plated into 96-well plates and treated as described under cell culture. At the end of the incubation period for 12 h with CO-RMs on day 3, an equal volume of the luminescent substrate and lysis buffer mix from the assay kit was added. The mixture was transferred to an opaque 96-well plate, and luminescence was recorded using a GloMax Microplate Luminometer (Promega). The index of cellular viability was calculated as the percentage of luminescence with respect to untreated control cells.

### Simultaneous Determination of Intracellular Total ROS Generation and Cell Death

ROS production and cell death were measured simultaneously by flow cytometry, allowing measurement of ROS production in gated viable cells. Carboxy-H_2_DCFDA is a cell-permeable ROS indicator that is non-fluorescent until the acetate groups are removed by intracellular esterases and oxidation occurs within the cell. When oxidized by various active oxygen species, it is irreversibly converted to the fluorescent form, DCF. The fluorescence generated by DCF is proportional to the rate of carboxy-H_2_DCFDA oxidation, which is in turn indicative of the cellular oxidizing activity and intracellular ROS levels. The cells were loaded in the dark with 10 μM carboxy-H_2_DCFDA for 40 min before the end of the treatment period with a given oxidant stimulus. The floating and adherent cells were collected by trypsinization and washed twice with HBSS with calcium and magnesium. Sytox Red (2.5 nM) dead cell stain was added to the cell suspension, and simultaneous detection of ROS production and cell death was performed in a single experimental setup by flow cytometry using 488 nm excitation wavelength with 530/30 nm (FL1; DCF) and 670/30 (FL4; Sytox Red) emission filters. Viable (Sytox Red-negative) cells were gated, and the green fluorescence of the ROS probe was analyzed in these cells.

### Simultaneous Determination of Mitochondrial $O_{2}^{•–}$ Anion and Cell Death

MitoSOX Red was used to detect mitochondrial $O_{2}^{•–}$ production. This modified cationic dihydroethidium dye localizes to the mitochondria where it is oxidized by $O_{2}^{•–}$ to generate bright red fluorescence (Robinson et al., 2006). Mitochondrial $O_{2}^{•–}$ generation and cell death were determined in a single experimental setup by treating the cell samples with MitoSOX Red and Sytox Red. The cells were loaded in the dark with 5 μM MitoSOX Red for 30 min before the end of the treatment period with a given oxidant stimulus, collected, washed twice with HBSS, and then stained with 2.5 nM Sytox Red. The samples were run on a flow cytometer with 488 nm excitation to measure oxidized MitoSOX Red in the FL2 channel and Sytox Red in the FL4 channel. Cell debris with low FSC (forward scatter) and SSC (side scatter) was excluded from the analysis. The mean fluorescence intensity (MFI) of MitoSOX Red staining was analyzed in the gated viable cell population (Sytox Red-negative). Thus, MitoSOX Red of the cells analyzed excluded any non-specific interference from dead cells.

For all experiments with analysis of ROS, (a) the fluorescence properties of at least 30,000 cells were acquired from each sample; (b) the samples were analyzed immediately after ending the incubation with the ROS indicator and strictly protected from light; (c) basal ROS generation in cells not exposed to TNF-α/CHX, H_2_O_2_, rotenone or antimycin-A, in culture medium alone or in medium containing the solvent of the drug to be studied vs. the oxidative stress stimulus, was used as a control; and (d) the MFIs of treated samples were expressed as percentage of the control MFI (set as 100%).

### Measurement of Mitochondrial Dysfunction

Determination of respiratory chain damage was performed by double staining with two different mitochondria-specific dyes, MitoTracker Green FM and MitoTracker Deep Red FM, to distinguish total and respiring mitochondria, respectively. Mitochondria in cells stained with MitoTracker Green FM dye exhibit bright green fluorescein-like fluorescence (FL1; fluorescence emission at 516 nm) as this dye accumulates in the lipid environment of mitochondria regardless of Ψ_m_ and becomes fluorescent. MitoTracker Deep Red FM does not fluoresce until it enters an actively respiring cell, where it is oxidized to the corresponding red fluorescence probe (FL4; fluorescence emission at 665 nm) that then sequesters in the mitochondria in proportion to Ψ_m_ (Cottet-Rousselle et al., 2011; Tang et al., 2011). So, MitoTracker Green will stain all mitochondria while MitoTracker Deep Red FM will stain active respiring mitochondria. The treated cells were incubated with 200 nM MitoTracker Green FM and 25 nM MitoTracker Deep Red FM in the dark at 37°C for 30 min before the end of the treatment period. Next, the cells were harvested and the pellets were suspended in 0.5 mL of PBS. The samples were analyzed immediately by flow cytometry. The percentage of MitoTracker Green-positive/MitoTracker Deep Red-negative cells is an important parameter of accumulation of cells with non-respiring (dysfunctional) mitochondria (Zhou et al., 2011).

### Simultaneous Determination of Mitochondrial Membrane Potential (Ψ_m_) and Cell Death

Determination of the changes in mitochondrial membrane potential (Ψ_m_) was performed using TMRM along with staining of Sytox Green (for cell death). TMRM is a cell-permeant, lipophilic cationic, red–orange fluorescent dye that is readily sequestered by active mitochondria. TMRM is a single wavelength dye that can be combined with a cell death marker to measure fluorescence exclusively in the viable cells. Briefly, following the treatment, the cells were washed twice with HBSS and then incubated with 200 nM TMRM solution prepared in DMEM medium for 30 min. Subsequently, the cells were washed twice with HBSS and stained with 2 nM Sytox Green prior to measurement. The percentage of TMRM and Sytox Green stained cells was calculated from at least 30,000 cells of each sample in comparison to the control. TMRM was excited at 488 nm, and fluorescence emitted at 588 nm (FL2) was measured in gated viable (Sytox Green-negative) cells by flow cytometry.

For assays of ROS, mitochondrial dysfunction and Ψ_m_, the samples were acquired using a BD LSR II flow cytometer and analyzed using FACSDiva software (BD Biosciences, San Jose, CA, USA).

### Statistical Analysis

All data are expressed as the mean ± SEM. The comparison of the means was performed using Student’s *t*-test for two groups of data and ANOVA followed by Bonferroni’s multiple comparison test for comparison of more than two groups. Differences were considered significant at *P* < 0.05.

## Results

### Effects of CORM-2 and CORM-401 on TNF-α/CHX-Induced Changes in Intracellular Total ROS Production and Cell Death

**FIGURE 1:**
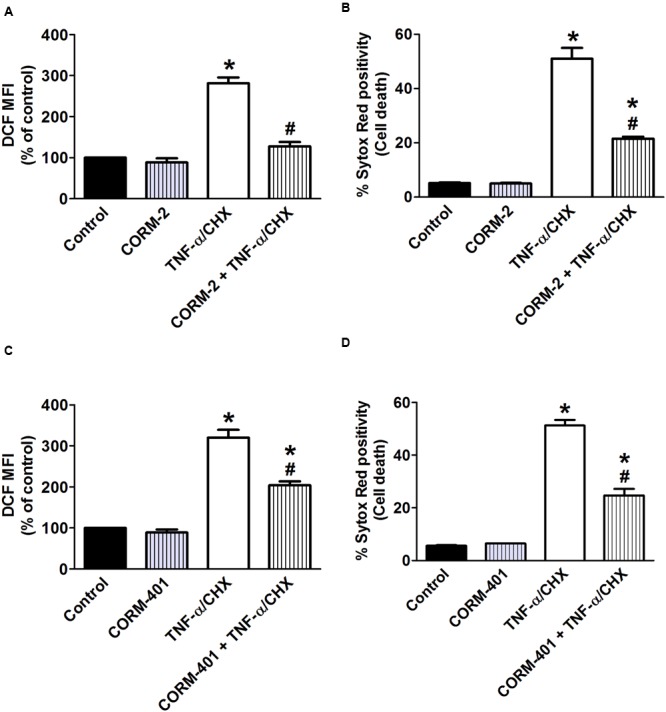
Effects of CORM-2 **(A,B)** or CORM-401 **(C,D)** on TNF-α/CHX-induced intracellular total ROS production and cell death in MODE-K cells. MODE-K cells were treated for 6 h with TNF-α (1 ng/ml) plus CHX (10 μg/ml) in the absence or presence of CORM-2 (40 μM) or CORM-401 (50 μM), added 1 h before TNF-α/CHX. Intracellular total ROS levels are assessed with carboxy-H_2_DCFDA (DCF), and gated on the viable, Sytox Red-negative population. Intracellular total ROS levels were expressed as % of control DCF MFI; cell death is expressed as % Sytox Red positivity. Control cells were incubated with serum-free medium alone; the effect of CORM-2 or CORM-401 *per se* was also tested. Mean ± SEM of three independent experiments. ^∗^*P* < 0.05 vs. control. ^#^*P* < 0.05 vs. TNF-α/CHX alone.

To determine whether CORM-2 and CORM-401 could modulate ROS production and exert cytoprotection during inflammatory conditions, TNF-α/CHX-induced intracellular total ROS production and cell death were simultaneously measured by flow cytometry. Treatment of MODE-K cells with TNF-α/CHX for 6 h increased total ROS by approximately 200% (**Figures 1A,C**) and induced approximately 50% cell death (**Figures 1B,D**). Treatment of MODE-K cells with CORM-2 *per se* was without effect, but CORM-2 abolished TNF-α/CHX-induced ROS production (**Figure 1A**) with a significant reduction of cell death (**Figure 1B**); iCORM-2 had no effect *per se* nor did it influence the TNF-α/CHX-induced changes (data not shown). CORM-401 *per se* did not affect basal total ROS production and cell survival. Treatment of cells with CORM-401 significantly reduced both TNF-α/CHX-induced ROS production and cell death (**Figures 1C,D**).

### Effects of CORM-2 and CORM-401 on H_2_O_2_-Induced Changes in Intracellular Total ROS Production

**FIGURE 2:**
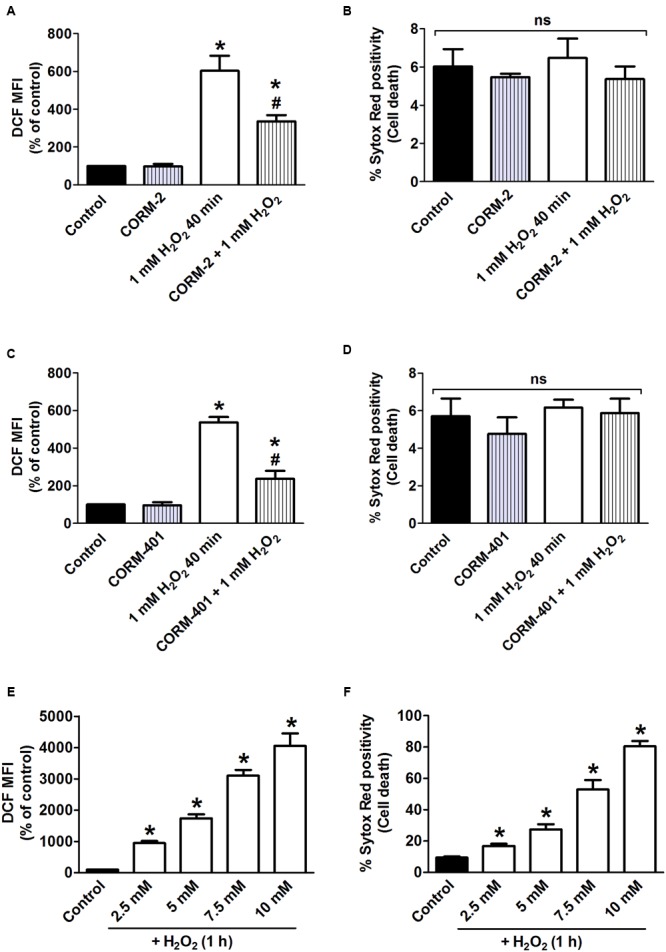
**(A–D)** Effects of CORM-2 **(A,B)** or CORM-401 **(C,D)** on H_2_O_2_-induced intracellular total ROS production and cell death in MODE-K cells. MODE-K cells were treated for 40 min with H_2_O_2_ (1 mM) in the absence or presence of CORM-2 (40 μM) or CORM-401 (50 μM), added 1 h before H_2_O_2_. Intracellular total ROS levels were assessed with carboxy-H_2_DCFDA (DCF), and gated on the viable, Sytox Red-negative population. Intracellular total ROS levels are expressed as % of control DCF MFI; cell death is expressed as % Sytox Red positivity. Control cells were incubated with serum-free medium alone; the effect of CORM-2 or CORM-401 *per se* was also tested. Mean ± SEM of three independent experiments. ^∗^*P* < 0.05 vs. control. ^#^*P* < 0.05 vs. H_2_O_2_ alone. **(E,F)** Influence of H_2_O_2_ on intracellular total ROS production and cell death, measured as described for **(A–D)**. MODE-K cells were treated for 1 h with 2.5–10 mM H_2_O_2_. Control cells were incubated with serum-free medium alone. Mean ± SEM of three independent experiments. ^∗^*P* < 0.05 vs. control.

The capacity of CORM-2 and CORM-401 to modulate oxidant-generating system(s) was further investigated *in vitro* using the model of H_2_O_2_-induced oxidative stress in MODE-K cells. First, incubation of cells with 1 mM H_2_O_2_ for 40 min was studied, as this condition was previously shown to induce a level of intracellular total ROS production relatively similar to that induced by TNF-α/CHX treatment for 6 h, albeit without inducing cell death (Babu et al., 2015b). H_2_O_2_ increased ROS production by 430–505% compared with control (**Figures 2A,C**) indeed without inducing cell death (**Figures 2B,D**). Both CORM-2 and CORM-401 significantly attenuated the H_2_O_2_-induced increase in total ROS production (**Figures 2A,C**).

As the release of CO from CORM-401 has been shown to be accelerated by increasing concentrations of oxidants, a concentration-response curve to H_2_O_2_ higher than 1 mM was constructed, assessing both intracellular total ROS production and cell death (**Figures 2E,F**). From these experiments, incubation of cells with 7.5 mM H_2_O_2_ for 1 h was selected as this condition induced a level of cell death similar to that induced by TNF-α/CHX for 6 h, i.e., 57 ± 4% (**Figure 2F**); this concentration of H_2_O_2_ increased ROS production by 2900% relative to the control (**Figure 2E**).

**FIGURE 3:**
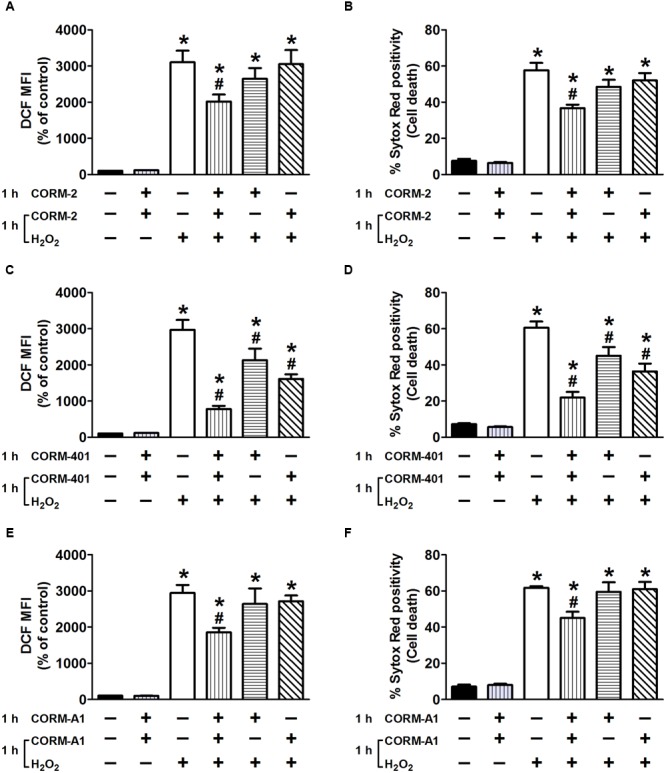
Effects of CORM-2 **(A,B)**, CORM-401 **(C,D)** or CORM-A1 **(E,F)** on H_2_O_2_-induced intracellular total ROS production and cell death in MODE-K cells. MODE-K cells were treated for 1 h with H_2_O_2_ (7.5 mM); CORM-2 (40 μM), CORM-401 (50 μM) or CORM-A1 (100 μM) was either pre-incubated for 1 h and then co-incubated with H_2_O_2_ for 1 h (vertical striped bars), only pre-incubated for 1 h (horizontal striped bars) or co-incubated with H_2_O_2_ for 1 h (transverse striped bars). Intracellular total ROS levels were assessed with carboxy-H_2_DCFDA (DCF), and gated on the viable, Sytox Red-negative population. Intracellular total ROS levels are expressed as % of control DCF MFI; cell death is expressed as % Sytox Red positivity. Control cells were incubated with serum-free medium alone; the effect of CORM-2, CORM-401 or CORM-A1 *per se* was also tested. Mean ± SEM of three independent experiments. ^∗^*P* < 0.05 vs. control. ^#^*P* < 0.05 vs. H_2_O_2_ alone.

When the standard treatment protocol was used for CORM-2 (i.e., pre-treatment for 1 h before exposure to H_2_O_2_ and then co-treatment during exposure to H_2_O_2_), H_2_O_2_-induced ROS production and cell death were significantly reduced by 34 ± 10% and 41 ± 4%, respectively (*n* = 3; **Figures 3A,B**). By contrast, either pre-treatment with CORM-2 for 1 h or co-treatment with CORM-2 during the 1 h exposure to H_2_O_2_ did not affect H_2_O_2_-induced ROS production (**Figure 3A**) or cell death (**Figure 3B**). Pre-treatment of cells with CORM-401 followed by co-treatment with CORM-401 and H_2_O_2_ was even more effective as it decreased both H_2_O_2_-induced ROS production and cell death by 75 ± 5% and 72 ± 6%, respectively (*n* = 3; **Figures 3C,D**). Interestingly, and in contrast to the observations made with CORM-2, just pre-treating cells with CORM-401 for 1 h or just co-treating them with CORM-401 during the 1 h exposure to H_2_O_2_ was sufficient to significantly reduce H_2_O_2_-induced ROS production and cell death, although to a lesser extent compared with the pre- plus co-treatment protocol (*n* = 3; **Figures 3C,D**). Another water-soluble CO-RM, CORM-A1 showed similar effects as CORM-2 (*n* = 3; **Figures 3E,F**).

### Effects of CORM-2 and CORM-401 on TNF-α/CHX-Induced Changes in Mitochondrial $O_{2}^{•–}$ Production and Cell Death

**FIGURE 4:**
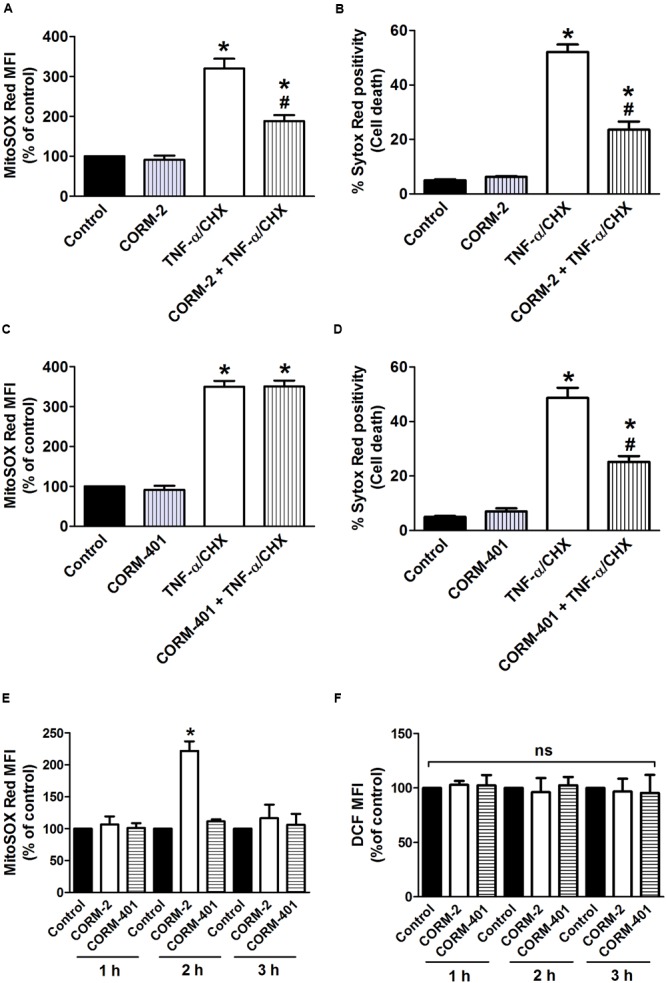
**(A–D)** Effects of CORM-2 **(A,B)** or CORM-401 **(C,D)** on TNF-α/CHX-induced mitochondrial $O_{2}^{•–}$ production and cell death in MODE-K cells. MODE-K cells were treated for 6 h with TNF-α (1 ng/ml) plus CHX (10 μg/ml) in the absence or presence of CORM-2 (40 μM) or CORM-401 (50 μM), added 1 h before TNF-α/CHX. Mitochondrial $O_{2}^{•–}$ levels were assessed with MitoSOX Red, and gated on the viable, Sytox Red-negative population. Mitochondrial $O_{2}^{•–}$ levels are expressed as % of control MitoSOX Red MFI; cell death is expressed as % Sytox Red positivity. Control cells were incubated with serum-free medium alone; the effect of CORM-2 or CORM-401 *per se* was also tested. Mean ± SEM of three independent experiments. ^∗^*P* < 0.05 *vs.* control. ^#^*P* < 0.05 vs. TNF-α/CHX alone. **(E,F)** Influence of CORM-2 (40 μM) or CORM-401 (50 μM) *per se*, incubated for 1–3 h, on levels of mitochondrial $O_{2}^{•–}$ **(E)**, measured as described for (**A–D)**, and intracellular total ROS **(F)**, measured as described in **Figure 1**. Mean ± SEM of three independent experiments. ^∗^*P* < 0.05 vs. untreated control at the given time point.

The influence of CORM-2 and CORM-401 on TNF-α/CHX-induced mitochondrial $O_{2}^{•–}$ production was assessed by using the fluorescent probe MitoSOX Red in conjunction with Sytox Red, to discriminate viable vs. dead cells, in a single experimental setup. Exposure of cells to TNF-α/CHX increased mitochondrial $O_{2}^{•–}$ production by approximately 200% relative to the control, and treatment with CORM-2 reduced this effect (**Figure 4A**) alongside a concomitant decrease in TNF-α/CHX-induced cell death (**Figure 4B**). iCORM-2 had no effect *per se* nor did it influence the TNF-α/CHX-induced changes (data not shown). By contrast, although CORM-401 significantly reduced TNF-α/CHX-induced cell death (**Figure 4D**), it did not influence TNF-α/CHX-induced mitochondrial $O_{2}^{•–}$ production (**Figure 4C**).

Incubated for 7 h, neither CORM-2 nor CORM-401 *per se* increased mitochondrial $O_{2}^{•–}$ production (**Figures 4A,C**). When treating the cells for 1, 2, or 3 h, CORM-2 promoted a significant increase in mitochondrial $O_{2}^{•–}$ when incubated for 2 h, whereas CORM-401 did not change mitochondrial $O_{2}^{•–}$ production (**Figure 4E**); also iCORM-2 was without effect (data not shown). Treatment with CORM-2 or CORM-401 did not result in an increase in intracellular total ROS production at these time points (**Figure 4F**).

### Effects of CORM-2 and CORM-401 on Mitochondrial Complex I- and Complex III-Induced Changes in Mitochondrial $O_{2}^{•–}$ and Cell Death

**FIGURE 5:**
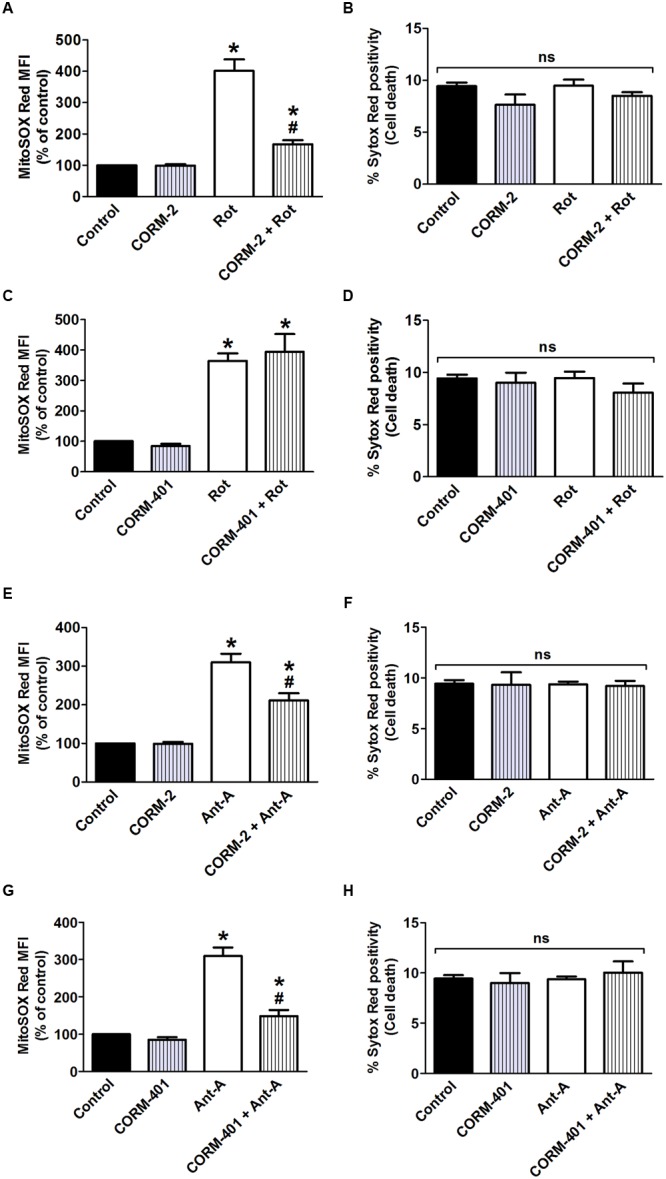
Effects of CORM-2 or CORM-401 on mitochondrial complex I (rotenone; Rot; **A–D**)- and mitochondrial complex III (antimycin-A; Ant-A; **E–H**)-induced mitochondrial $O_{2}^{•–}$ production and cell death in MODE-K cells. MODE-K cells were treated for 6 h with rotenone (7.5 μM) or antimycin-A (10 μM) in the absence or presence of CORM-2 (40 μM; **A,B,E,F**) or CORM-401 (50 μM; **C,D,G,H**), added 1 h before rotenone or antimycin-A. Mitochondrial $O_{2}^{•–}$ levels were assessed with MitoSOX Red, and gated on the viable, Sytox Red-negative population. Mitochondrial $O_{2}^{•–}$ levels are expressed as % of control MitoSOX Red MFI; cell death is expressed as % Sytox Red positivity. Control cells were incubated with serum-free medium alone; the effect of CORM-2 or CORM-401 *per se* was also tested. Mean ± SEM of three independent experiments. ^∗^*P* < 0.05 vs. control. ^#^*P* < 0.05 vs. rotenone or antimycin-A alone.

Rotenone (7.5 μM), an inhibitor of mitochondrial complex I, induced an increase in mitochondrial $O_{2}^{•–}$ level comparable to that induced by TNF-α/CHX (**Figures 5A,C**), without affecting cell survival (**Figures 5B,D**). The concentration of 7.5 μM rotenone was selected based on a concentration-response study of the effect of rotenone on mitochondrial $O_{2}^{•–}$, when incubated for 6 h to mimic the exposure time to TNF-α/CHX (Babu et al., 2015a). Treatment with CORM-2 nearly abolished rotenone-induced mitochondrial $O_{2}^{•–}$ production, whereas CORM-401 had no effect (**Figures 5A,C**).

The influence of CORM-2 and CORM-401 on mitochondrial $O_{2}^{•–}$ production at the level of mitochondrial complex III was investigated by use of the complex III inhibitor antimycin-A, an agent well known to induce $O_{2}^{•–}$ production at this site. Antimycin-A was used at 10 μM for 6 h (Babu et al., 2015a) as this concentration did not induce cell death in MODE-K cells (see **Figures 5F,H**), but increased mitochondrial $O_{2}^{•–}$ production to a level comparable to that induced by TNF-α/CHX (see **Figures 5E,G**). Both CORM-2 and CORM-401 significantly decreased (**Figures 5E,G**) antimycin-A-induced mitochondrial $O_{2}^{•–}$ production.

iCORM-2 had no influence on the rotenone- or antimycin-A-induced increase in mitochondrial $O_{2}^{•–}$ levels (data not shown).

### Effects of CORM-2 and CORM-401 on TNF-α/CHX-Induced Changes in Mitochondrial Dysfunction and Mitochondrial Membrane Potential (Ψ_m_) of MODE-K Cells

**FIGURE 6:**
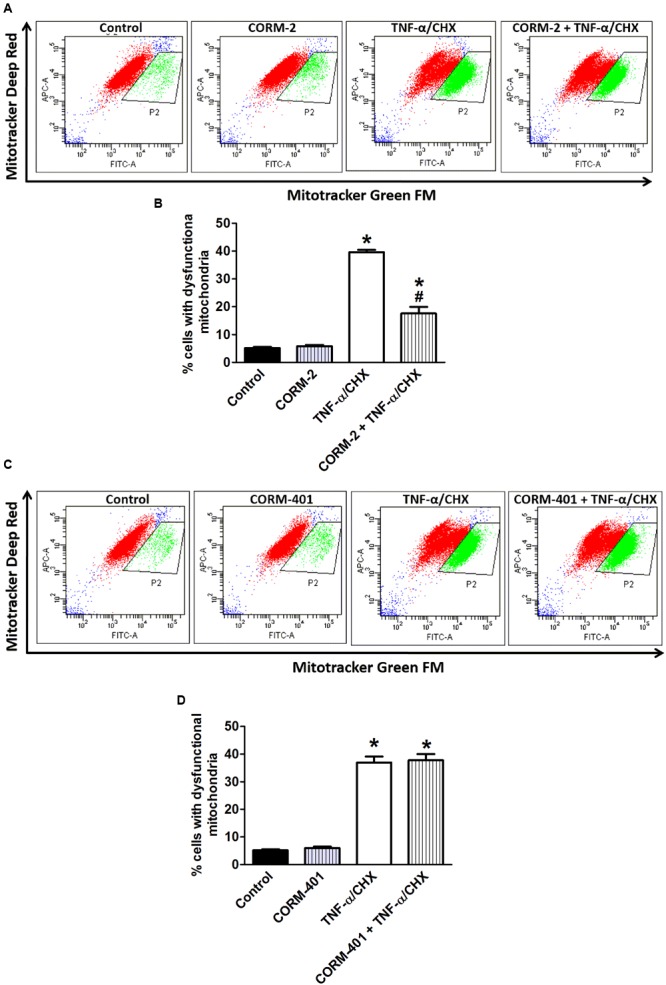
Effects of CORM-2 **(A,B)** or CORM-401 **(C,D)** on TNF-α/CHX-induced mitochondrial dysfunction in MODE-K cells. MODE-K cells were treated for 6 h with TNF-α (1 ng/ml)/CHX (10 μg/ml), in the absence and presence of CORM-2 (40 μM) or CORM-401 (50 μM), added 1 h before TNF-α/CHX. The number of cells with respiring mitochondria was assessed with MitoTracker Green FM (FL1; FITC) and MitoTracker Deep Red FM (FL4; APC). Control cells were incubated with serum-free medium alone; the effect of CORM-2 or CORM-401 *per se* was also tested. **(A,C)** Representative dot plots of the flow cytometric analysis showing the effect of CORM-2 **(A)** or CORM-401 **(C)** on MitoTracker Green FM (total mitochondria) vs. MitoTracker Deep Red FM (actively respiring mitochondria) fluorescence of viable cells after treatment with TNF-α/CHX. Cells in the gated region (P2) contain respiration-interrupted mitochondria. **(B,D)** Quantification of flow cytometric measurements (expressed as % of cells with dysfunctional mitochondria) for experiments with CORM-2 **(B)** and CORM-401 **(D)**. Mean ± SEM of three independent experiments. ^∗^*P* < 0.05 vs. control. ^#^*P* < 0.05 vs. TNF-α/CHX alone.

Double staining of MODE-K cells with two different mitochondria-specific dyes to distinguish actively respiring (MitoTracker Deep Red FM-positive) and total (MitoTracker Green FM-positive) mitochondria revealed an increase in the number of respiration-interrupted mitochondria in the P2-gated region upon treatment of cells with TNF-α/CHX (representative examples in the third panel of **Figures 6A,C**) compared with untreated control cells (35–40% vs. 4–6%; **Figures 6B,D**). Treatment with CORM-2 (**Figures 6A,B**) significantly attenuated the TNF-α/CHX-induced increase in dysfunctional mitochondria, whereas treatment with CORM-401 did not have any effect on this parameter (**Figures 6C,D**).

**FIGURE 7:**
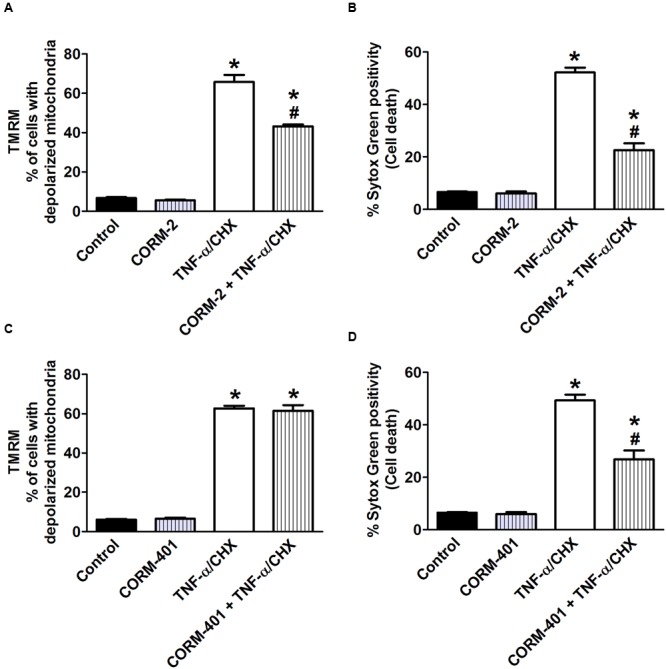
Effects of CORM-2 **(A,B)** or CORM-401 **(C,D)** on TNF-α/CHX-induced changes in mitochondrial membrane potential and cell death in MODE-K cells. MODE-K cells were treated for 6 h with TNF-α (1 ng/ml)/CHX (10 μg/ml), incubated for 6 h, in the absence and presence of CORM-2 (40 μM) or CORM-401 (50 μM), added 1 h before TNF-α/CHX. Mitochondrial membrane potential was assessed with TMRM, and gated on the viable, Sytox Green-negative population. Mitochondrial membrane potential is expressed as % of cells with depolarized mitochondria as measured by TMRM staining; cell death is expressed as % Sytox Green positivity. Control cells were incubated with serum-free medium alone; the effect of CORM-2 or CORM-401 *per se* was also tested. Mean ± SEM of three independent experiments. ^∗^*P* < 0.05 vs. control. ^#^*P* < 0.05 vs. TNF-α/CHX alone.

We next studied the influence of CORM-2 and CORM-401 on TNF-α/CHX-induced changes in Ψ_m_ using a potentiometric dye, TMRM. TMRM facilitates analysis of cell death simultaneously in combination with the cell death marker Sytox Green. Approximately 7–8% of control cells showed depolarized mitochondria. Treatment of cells with TNF-α/CHX caused approximately 60% depolarization of mitochondria (**Figures 7A,C**). CORM-2 decreased the number of depolarized cells by TNF-α/CHX to 39% (**Figure 7A**) but CORM-401 did not influence this parameter (**Figure 7C**), whereas their inhibitory effect on TNF-α/CHX-induced cell death was confirmed (**Figures 7B,D**).

iCORM-2 had no influence on the TNF-α/CHX-induced changes in both assays (data not shown).

## Discussion

TNF-α/CHX-induced apoptosis of MODE-K cells is associated with a marked production of ROS, the major sources being NOX and mitochondrial ETC complexes I and II (Babu et al., 2012, 2015b). One of the possible cellular mechanisms by which CO confers cytoprotection is via modulation of ROS production (Peers et al., 2015). We previously found that water-soluble CORM-A1 reduces TNF-α/CHX-induced apoptosis by inhibiting NOX-derived ROS production but not mitochondrial $O_{2}^{•–}$ production induced by TNF-α (Babu et al., 2012, 2015a); treatment of MODE-K cells with CORM-A1 at concentrations that display a cytoprotective effect did not change basal mitochondrial $O_{2}^{•–}$ production, even in the first hours after its administration. These results suggest that CORM-A1 is unable to interfere with sites of ROS production at the mitochondrial level; this result might be attributed to the water-soluble property of this CO-RM impairing its penetration into the cell and/or through the inner mitochondrial membrane (Horton et al., 2008). In the present study, we investigated the influence of the lipid-soluble CORM-2 under oxidative stress conditions in comparison with the newly developed water-soluble CORM-401, which was shown to release more equivalents of CO under oxidant conditions (Fayad-Kobeissi et al., 2016); see Table 2A for a qualitative summary of the effects of CORM-2 and CORM-401 in basal conditions and vs. TNF-α/CHX, rotenone, and antimycin-A treatment.

**Table 2a:** Qualitative summary of the effects of CORM-2 and CORM-401, incubated for 7 h, in basal conditions and vs. TNF-α/CHX, rotenone and antimycin-A treatment.

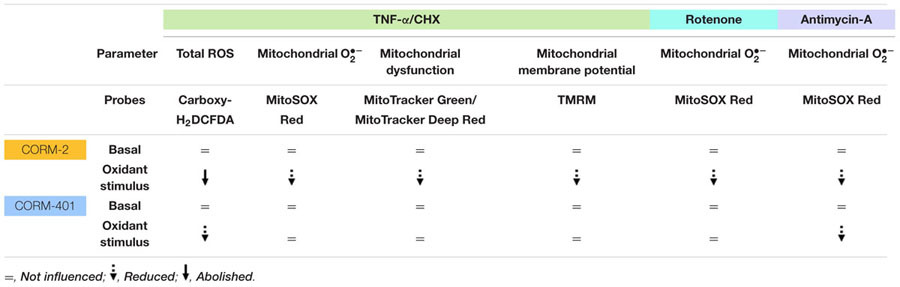

### Mechanism of Action of CORM-2 during TNF-α/CHX-Induced Oxidative Stress

In contrast to previous results with CORM-A1 (Babu et al., 2015a), pre- and co-incubation of MODE-K cells with CORM-2 abolished TNF-α/CHX-induced total cellular ROS production and markedly diminished cell death. CORM-2 also reduced TNF-α/CHX-induced mitochondrial $O_{2}^{•–}$ production suggesting that CO released from CORM-2 can interfere at mitochondrial ROS production sites. CORM-2 indeed also reduced rotenone and antimycin-A-induced mitochondrial $O_{2}^{•–}$ production demonstrating its capacity to mitigate complex I and complex III-derived ROS. The observed effects of CORM-2 can be attributed to the effect of CO released from it, as iCORM-2 both *per se* and in the presence of the stimuli (TNF-α/CHX, rotenone and antimycin-A) had no effect. Heme-containing proteins in mitochondria (cytochromes) and NOX enzymes in the cells are considered the major targets of CO due to the high affinity of CO for heme (Foresti and Motterlini, 2010). At the mitochondrial level, CO is known to inhibit complex IV (cytochrome c oxidase), the terminal enzyme within the ETC, resulting in a significant transient burst of mitochondria-derived ROS ($O_{2}^{•–}$). Several reports support that a partial inhibition of complex IV may create a preconditioning state to protect cells against subsequent oxidative insults (Chin et al., 2007; Zuckerbraun et al., 2007; Kim et al., 2008). CORM-2 *per se* increased mitochondrial $O_{2}^{•–}$ levels temporarily at 2 h of its incubation in MODE-K cells, whereas the total cellular ROS level measured at the same time point did not show any increase; the intracellular ROS detecting probe carboxy-H_2_DCFDA can indeed be oxidized by ROS types, such as H_2_O_2_ and hydroxyl radical but not $O_{2}^{•–}$ (Myhre et al., 2003). The early mitochondrial $O_{2}^{•–}$ induction by CORM-2 might contribute to its cytoprotective effect against TNF-α/CHX treatment. The induction of mitochondrial $O_{2}^{•–}$ production by CORM-2 *per se* and the reduction by CORM-2 of mitochondrial $O_{2}^{•–}$ produced upon TNF-α/CHX, rotenone and antimycin-A treatment might be related to its lipid solubility. It seems plausible that the hydrophobic surface of CORM-2 enables its easy penetration through the outer and inner mitochondrial membrane, thus releasing CO inside the matrix. CORM-2 increased mitochondria-derived ROS as early as 30 min after its incubation with human airway smooth muscle cells (Taille et al., 2005). In MODE-K cells, measurable mitochondrial $O_{2}^{•–}$ was observed only after 2 h incubation, but this does not exclude the induction of mitochondrial $O_{2}^{•–}$ by CORM-2 before 2 h. We have previously identified that significant ROS production by TNF-α in MODE-K cells can only be observed from 2 h on, in contrast to other IECs, indicating that MODE-K cells are able to counteract the initial burst of oxidative stress up to this time point, after which the antioxidant defense can no longer be maintained (Babu et al., 2015b). The results of the current study therefore suggest a role of the lipid-soluble nature of CORM-2 in interfering with mitochondrial ROS signaling in MODE-K IECs; further studies in other cell types are warranted to corroborate this phenomenon. Superoxide anion produced from NOX is converted into H_2_O_2_ by superoxide dismutase (SOD)-1 (Cu/ZnSOD) in the cytoplasm, whereas $O_{2}^{•–}$ produced from mitochondria are either released as such into the cytoplasm by voltage-dependent anion channels (VDACs) or first converted into H_2_O_2_ by SOD-2 (MnSOD)/SOD-1 before diffusing across the mitochondrial membrane into the cytoplasm (Han et al., 2003a). CORM-2 only partially reduced TNF-α/CHX-induced mitochondrial $O_{2}^{•–}$ production, so that some mitochondrial $O_{2}^{•–}$ /H_2_O_2_ theoretically might leak into the cytoplasm in MODE-K cells. However, at least H_2_O_2_ should then contribute to the ROS signal picked up with carboxy-H_2_DCFDA and this part should not be influenced by treatment with CORM-2, as originating from mitochondrial $O_{2}^{•–}$ not suppressed with CORM-2. As the increase in the DCF signal by TNF-α/CHX was fully abolished by CORM-2, this mitochondrial leakage does not seem to occur in MODE-K cells; this result further illustrates that CORM-2 abolishes NOX-derived ROS in MODE-K cells.

### Mechanism of Action of CORM-401 during TNF-α/CHX-Induced Oxidative Stress

Similar to CORM-A1, CORM-401 reduced TNF-α/CHX-induced total cellular ROS production and cell death without any influence on TNF-α/CHX-induced mitochondrial $O_{2}^{•–}$ production. Notably, CORM-401 *per se* did not have any effect on mitochondrial $O_{2}^{•–}$ production. CORM-A1 attenuated the decrease in Ψ_m_ and the increase in mitochondrial dysfunction by TNF-α/CHX, which might be related to its inhibitory effect on mitochondrial respiration (Babu et al., 2015a). By contrast, CORM-401 did not influence these mitochondrial effects mediated by TNF-α. The inability of CORM-401 to modulate TNF-α/CHX-induced mitochondrial $O_{2}^{•–}$ production and other mitochondrial parameters seems to exclude mitochondria as a possible target influenced by CORM-401 during TNF-α/CHX-induced cell death. Complexes I and II of the ETC are the major mitochondrial ROS production sites during TNF-α/CHX-induced cell death in MODE-K cells (Babu et al., 2015b). Consistent with the observation that CORM-401 does not influence TNF-α/CHX-induced mitochondrial $O_{2}^{•–}$, CORM-401 was not able to reduce mitochondrial $O_{2}^{•–}$ production by the complex I inhibitor rotenone. This result is probably related to the fact that rotenone-induced mitochondrial $O_{2}^{•–}$ is released into the mitochondrial matrix (Chen et al., 2003; Rodriguez-Rocha et al., 2013). Indeed, as reported for CORM-A1 (Babu et al., 2015a), CORM-401 was able to reduce antimycin-A-induced mitochondrial $O_{2}^{•–}$ generation. This result could be attributed to the fact that the mitochondrial $O_{2}^{•–}$ generated by the Qi site inhibitor of complex III antimycin-A is reported to be fully (St-Pierre et al., 2002) or at least partially (Han et al., 2003b) released into the mitochondrial intermembrane space. Still, we have previously observed that myxothiazole, an inhibitor of complex III at the Qo site, partially reduced TNF-α/CHX-induced total ROS and cell death in MODE-K cells (Babu et al., 2015b), implying that part of TNF-α/CHX-induced ROS must also be released into the mitochondrial intermembrane space. The lack of any effect of CORM-401 vs. TNF-α/CHX-induced mitochondrial ROS might be related to near full use of CORM-401-derived CO in the cytoplasm to counteract NOX-derived ROS upon exposure to TNF-α. As NOX enzymes are not activated by antimycin-A, a higher amount of CO from CORM-401 might reach the mitochondrial intermembrane space upon exposure of MODE-K cells to antimycin-A, resulting in a more pronounced reduction of mitochondrial $O_{2}^{•–}$ than observed with CORM-2 treatment. As CORM-401 does not interfere with TNF-α/CHX-induced mitochondrial ROS, its inhibitory action on total cellular ROS induced by TNF-α/CHX treatment is exclusively related to inhibition of ROS derived from NOX, the second major source of TNF-α/CHX-induced ROS production. Inhibition of NOX by CO leading to decreased cytoplasmic $O_{2}^{•–}$ production has been previously reported (Bilban et al., 2006; Srisook et al., 2006; Wang et al., 2007; Kelsen et al., 2008).

### Differential Effects of CORM-2, CORM-A1 and CORM-401 during H_2_O_2_-Induced Cytotoxic Oxidative Stress

The main cellular ROS involved in redox signaling is probably H_2_O_2_, as $O_{2}^{•–}$ produced by various intracellular sources is either spontaneously or through SODs dismutated into H_2_O_2_. H_2_O_2_ is the most abundant ROS in cells; it is relatively stable and less toxic than other types of ROS and capable of diffusing across membranes (Bienert et al., 2006). Physiologically, low endogenous levels of H_2_O_2_ function as signaling molecules for the regulation of eukaryotic signal transduction but endogenous overproduction of H_2_O_2_ is implicated in pathophysiological oxidative stress (Van de Bittner et al., 2010). Similarly, exogenous addition of higher concentrations of H_2_O_2_ leads to oxidative stress and apoptotic cell death (Veal et al., 2007). The degree of effect of CORM-2 and CORM-401 under oxidative stress conditions in comparison with CORM-A1 was therefore also investigated vs. H_2_O_2_-induced ROS in MODE-K cells (see Table 2B for a qualitative summary of the effects of CORM-2, CORM-401, and CORM-A1 vs. H_2_O_2_). Incubation of MODE-K cells with CORM-2 or CORM-401 from 1 h before and during exposure to a non-cytotoxic concentration of H_2_O_2_ (1 mM) for 40 min similarly reduced H_2_O_2_-induced intracellular total ROS levels, which is comparable to our earlier observation with CORM-A1 (Babu et al., 2015a). However, when tested vs. 7.5 mM H_2_O_2_ for 1 h, inducing a similar level of cell death as TNF-α/CHX, but a much higher degree of total ROS, differences were observed for CORM-401 vs. CORM-2 and CORM-A1. CORM-401 is a water-soluble CO-RM with a half-life of CO release of 12-14 min; it is able to release up to three CO per mole of compound, whereas CORM-2 and CORM-A1 release one CO per mole of compound. Moreover, the release of CO is increased over time in the presence of biologically relevant oxidants, such as H_2_O_2_ (Fayad-Kobeissi et al., 2016). This acceleration of CO release from CORM-401 when more oxidants are present probably explains why CORM-401 is active in the presence of 7.5 mM H_2_O_2_ when co-incubating the cells with the compound only during the 1 h exposure to H_2_O_2_; the high degree of oxidative stress imposed by 7.5 mM H_2_O_2_ can be expected to maximally accelerate CO release from CORM-401. This effect does not occur with CORM-2 and CORM-A1, which were ineffective with this treatment protocol. Surprisingly, CORM-401 was also mildly effective when only pre-incubated for 1 h before exposure of the cells to 7.5 mM H_2_O_2_. There is not a high ROS level in MODE-K cells before exposure to 7.5 mM H_2_O_2_; thus, CO release from CORM-401 is not accelerated. Still, the amount of CO released from CORM-401 seems sufficient to provide some protection to the cells against 7.5 mM H_2_O_2_ after washout of CORM-401. We do not have a definitive explanation for this observation, which did not occur with CORM-2 and CORM-A1. Both these compounds were only effective vs. 7.5 mM H_2_O_2_ upon 1 h pre-incubation plus 1 h co-incubation. As no real iCORM-401 can be prepared, some influence of the scaffold of CORM-401 cannot fully be excluded.

**Table 2b:** Qualitative summary of the effects of CORM-2, CORM-401 and CORM-A1 vs. H_2_O_2_ treatment (1 mM for 40 min and 7.5 mM for 1 h).

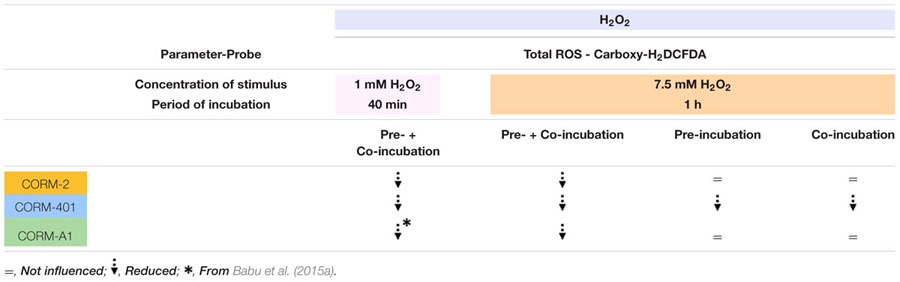

## Conclusion

CORM-2 and CORM-401 show differential cytoprotective effects under oxidative stress conditions in MODE-K IECs. Both CORM-2 and CORM-401 show antioxidant and cytoprotective effects under oxidative stress associated with inflammation (TNF-α/CHX). The cytoprotective effect of CORM-401 mitigates NOX-derived ROS, whereas CORM-2 interferes with both NOX and mitochondria-derived ROS to protect MODE-K cells from TNF-α/CHX-induced cell death; the mitochondrial effect of CORM-2 might be related to its lipophilicity. CORM-401 was more effective than CORM-2 in protecting cells against oxidative stress and cell death, induced by a high concentration of exogenous H_2_O_2_. This result might be related to the ability of CORM-401 to release more CO under oxidative conditions, suggesting that this compound may be effective under conditions of persistent oxidative stress such as in the case of acute and chronic GI disorders. Once probes are available allowing to analyze the subcellular location of released CO, it will be possible to investigate whether CO released from different CO-RMs can show a different subcellular trajectory.

## Author Contributions

DB and RL conceived and designed the experiments. DB performed the experiments. GL and RM contributed to the reagents, analytical tools and revision of the manuscript. DB and RL wrote the manuscript. All authors read and approved the final manuscript.

## Conflict of Interest Statement

The authors declare that the research was conducted in the absence of any commercial or financial relationships that could be construed as a potential conflict of interest.
